# Determining the rotational alignment of the tibial component referring to the tibial tubercle during total knee arthroplasty: the tibial tubercle–trochlear groove can be an aid

**DOI:** 10.1186/s13018-022-03139-9

**Published:** 2022-05-04

**Authors:** He Zhang, Chengming Cao, Han Zhang, Shoujiang Han

**Affiliations:** 1 Handan Branch, Huabeiyiliao Jiankangjituan Fengfeng Zongyiyuan, Handan, 056000 Hebei China; 2 Department of Orthopaedic Surgery, Huabeiyiliao Jiankangjituan Fengfeng Zongyiyuan, Handan, 056000 Hebei China

**Keywords:** Total knee arthroplasty, Akagi line, Insall line, Tibial tubercle-to-trochlear groove distance, Rotational alignment

## Abstract

**Background:**

There is no consensus on anatomic landmarks or reference axes with which to accurately align rotational position of tibial component. Using the tibial tubercle, commonly referring to the Akagi line and the Insall line, for anatomic reference was widely accepted. However, it is unknown about the predictors that may affect the reliability of using the tibial tubercle for aligning tibial component rotation. The aims of our study were (1) to investigate the reproducibility and accuracy of using the tibial tubercle for aligning tibial component rotation and (2) to determine predictors resulting in discrepancies of the tibial component rotation when referring to the tibial tubercle.

**Method:**

A total of 160 patients with osteoarthritis were recruited before total knee arthroplasty. The angle *α* formed by the tibial anteroposterior (AP) axis and the Akagi line and the angle *β* formed by the tibial AP axis and the Insall line were measured to quantify the discrepancies of the Akagi line and the Insall line. Independent variables, including the tibial tubercle-to-trochlear groove distance (TT-TG), tibial tubercle to posterior cruciate ligament (TT-PCL), and knee rotation angle (KRA), hip–knee–ankle angle (HKA), medial proximal tibial angle (MPTA), and tibial bowing (TB), were measured. Pearson’s product moment correlation coefficients and multivariable linear regression analysis were calculated to assess relationships between independent variables and the two defined angles.

**Results:**

All defined measurement were available for 140 patients. The Akagi line rotated internally with 1.03° ± 4.25° in regard to the tibial AP axis. The Insall line rotated externally in regard to the tibial AP axis with 7.93° ± 5.36°. Three variables, including TT-TG, TT-PCL, and KRA, tended to be positively correlated with the angle *α* and the angle *β*. In terms of a cutoff of TT-TG = 9 mm, 100% cases and 97% cases for using the Akagi line and Insall line, respectively, were located in the defined safe zone (− 5° to 10°).

**Conclusion:**

The tibial tubercle (the Akagi line and Insall line) is found to be a useful and promising anatomic landmark for aligning the tibial component rotation. The TT-TG, with a cutoff value of 9 mm, is helpful to choose the Akagi line or Insall line, alternatively.

## Introduction

Total knee arthroplasty (TKA) is one of the most common elective orthopedic surgeries performed worldwide and has been recognized as the highly cost-effective and curative method to relieve pain and improve function for patients with end-stage knee osteoarthritis when conservative treatment is unsuccessful [25, 36]. Over 95% of 10-year survivorship of primary TKA was reported in the investigation [33].

Besides restoration of the coronal and sagittal alignment of the affected lower extremity, proper femoral and tibial component rotational positioning is also essential for successful TKA. The consequences of component malrotation are considered to produce abnormal patellofemoral kinematics [2, 35, 47], and flexion and mid-flexion instability [6, 28], ultra-high molecular weight polyethylene wear [35, 49], stiffness [43], and abnormal gait patterns [48]. Femoral rotational reference axes, including the posterior condylar axis [34], the clinical and surgical trans-epicondylar axis [29], and Whiteside line [51], have been proved to be reproducible and reliable for aligning the femoral component rotation. Above all, the surgical trans-epicondylar axis (TEA) was believed to present the functional flexion–extension axis of knee and to be the optimum reference axis for decreasing patellofemoral and tibiofemoral complications [9, 12, 15, 31]. Unfortunately, the TEA could not be directly projected on tibial plateau during TKA.

Unlike the femoral side, there is no consensus on anatomic landmarks or reference axes with which to accurately align rotational position of tibial baseplate. High rates of malalignment greater than 3° were reported to occur in rotational position of tibial component [24, 32]. To acquire correct rotational orientation of the tibial component, numerous studies have attempt to propose intra- and extra-articular anatomic landmarks, including the tibial tubercle [4, 13, 19], the posterior tibial condylar line [34], the mid-sulcus of the tibial spine [40], the tibial transcondylar line [52], and the axis of the second metatarsus bone [27]. Of these aforementioned anatomic landmarks, the tibial tubercle has been preferable for the majority of authors, since other references may be susceptible to morphological asymmetry, osteophyte formation, and deformity of the tibial plateau [27, 30]. The medial one-third of the tibial tubercle, initially introduced by Insall et al., has been as an anatomic landmark for rotational alignment of tibial component [19, 38, 39]. Dalury et al. suggested a line drawn 1 mm medial to the medial border of the tibial tubercle and going through the mid-sulcus of the tibial spines in 2001 [13], and then modified the line with 3–4 mm lateral to the tubercle's medial border in 2016 [14]. Akagi et al. described an alignment axis connecting the middle of the posterior cruciate ligament (PCL) to the medial border of the patellar tendon at the attachment level [4]. Kawahara et al. introduced the medial sixth of the patellar tendon at the attachment level as a useful anterior reference in rotational alignment of the tibial component [21]. Despite intensive concern and preference of the tibial tubercle, several studies doubt about its reproducibility and accuracy due to the variability of location and shape of the tibial tubercle [10, 19, 44]. However, there are no corresponding studies concerning about the predictors that may affect the reliability of using the tibial tubercle for aligning tibial component rotation until now.

The two basic objectives in this study were (1) to investigate the reproducibility and accuracy of using the tibial tubercle for aligning tibial component rotation and (2) to determine predictors resulting in discrepancies of the tibial component rotation when referring to the tibial tubercle.

## Methods

The study was approved by the Huabeiyiliao Jiankangjituan Fengfeng Zongyiyuan (ID: 2018-049-11). Informed consent was obtained from all patients before enrolled in the study.

### Participants

This team successively reviewed, from January 2018 to October 2019, medical records and imaging documents of 160 patients diagnosed with severe knee osteoarthritis and prior to primary TKA in our institute. Patients were excluded if any of these following items were met: (a) rheumatoid arthritis or inflammatory arthritis else; (b) surgical history of the affected lower extremities; (c) unavailable imaging modalities or the scheduled anatomic landmarks could not be distinctly identified; and (d) instability of knee joints or knee flexion contractures ≥ 10°.

### Radiology protocol

All patients had finished CT scans of the affected knee and standard standing AP radiographs of the entire lower extremities. The patients were placed in the supine position on the scanning table the knee fully extended with neutral or slight external rotation as needed for comfort. Straps were wrapped around the thigh and lower leg to minimize motion. A 16-detector row CT scanner (SOMATOM Sensation 16; Siemens Medical Solutions, Erlangen, Germany) was used. These CT scans were acquired using the following parameters: 512 × 512 matrix, 120 kV, 100 mAs, 1 s rotation time, 1 mm slice thickness, 0 mm slice skip, a 14 cm field of view, and bone kernel. The CT images obtained were then imported into a personal computer to carry out our measurements using RadiAnt DICOM software (Medical Ltd., Poznan, Poland) with an accuracy of 0.1° and 0.1 mm. This system allows linear and angular measurements to be made on images and marked while scrolling through successive axial CT images. The AP radiographs were performed in standing position.

### Measurements

To analyze risk factors that might affect the accuracy and reliability of the tibial tubercle used as a landmark for rotationally positioning the tibial component, this investigation reviewed axial measurements, including the tibial tubercle-to-trochlear groove distance (TT-TG), tibial tubercle to posterior cruciate ligament (TT-PCL), and knee rotation angle (KRA), and coronal measurements, including hip–knee–ankle angle (HKA), medial proximal tibial angle (MPTA), and tibial bowing (TB). Two authors, one well-trained orthopedist and one specialist in musculoskeletal radiology, independently performed all measurements in a blinded and randomized fashion. A subset of 30 patients was randomly selected to conduct all measurements again after four weeks.

### The discrepancies of the Akagi line and the Insall line

**Fig. 1:**
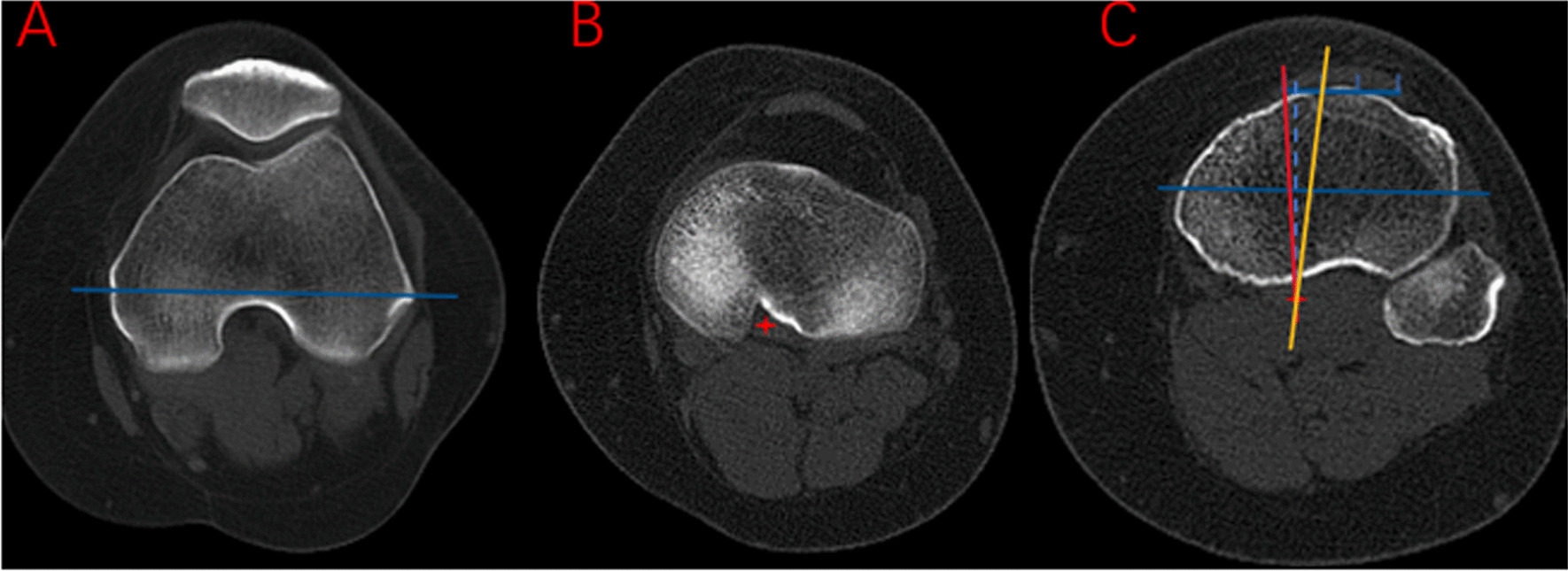
**a** TEA, the line connecting the sulcus of the medial epicondyle to the lateral epicondyle (blue line); **b** center of the PCL (red asterisk); **c** Angle *α*, formed by the tibial AP axis (dotted line) and the Akagi line (red line), angle *β*, formed by the tibial AP axis (dotted line) and the Insall line (yellow line). TEA, trans-epicondylar axis; PCL, posterior cruciate ligament; AP, anteroposterior

The TEA, connecting the sulcus of the medial epicondyle to the lateral epicondyle, was primarily drawn and then was projected onto the nearest plane of the tibial plateau, where the PCL could be clearly defined in the posterior condylar notch. The tibial anteroposterior (AP) axis was determined as the vertical line of the TEA, passing through the center of the PCL. Two points, locating at the medial border and the medial third of the patellar tendon, were labeled on the level of the most cephalad image on which the patellar tendon was completely in contact with the tibial tubercle, and then were cast on the plane of the tibial AP axis. The Akagi line and the Insall line were determined connecting the center of the PCL and the medial border and the medial third of the patellar tendon. The angle *α* formed by the tibial AP axis and the Akagi line and the angle *β* formed by the tibial AP axis and the Insall line were used to quantify the discrepancies of the Akagi line and the Insall line (Fig. 1). A positive value was adopted while the defined lines were externally rotated relative to the tibial AP axis.

**Fig. 2:**
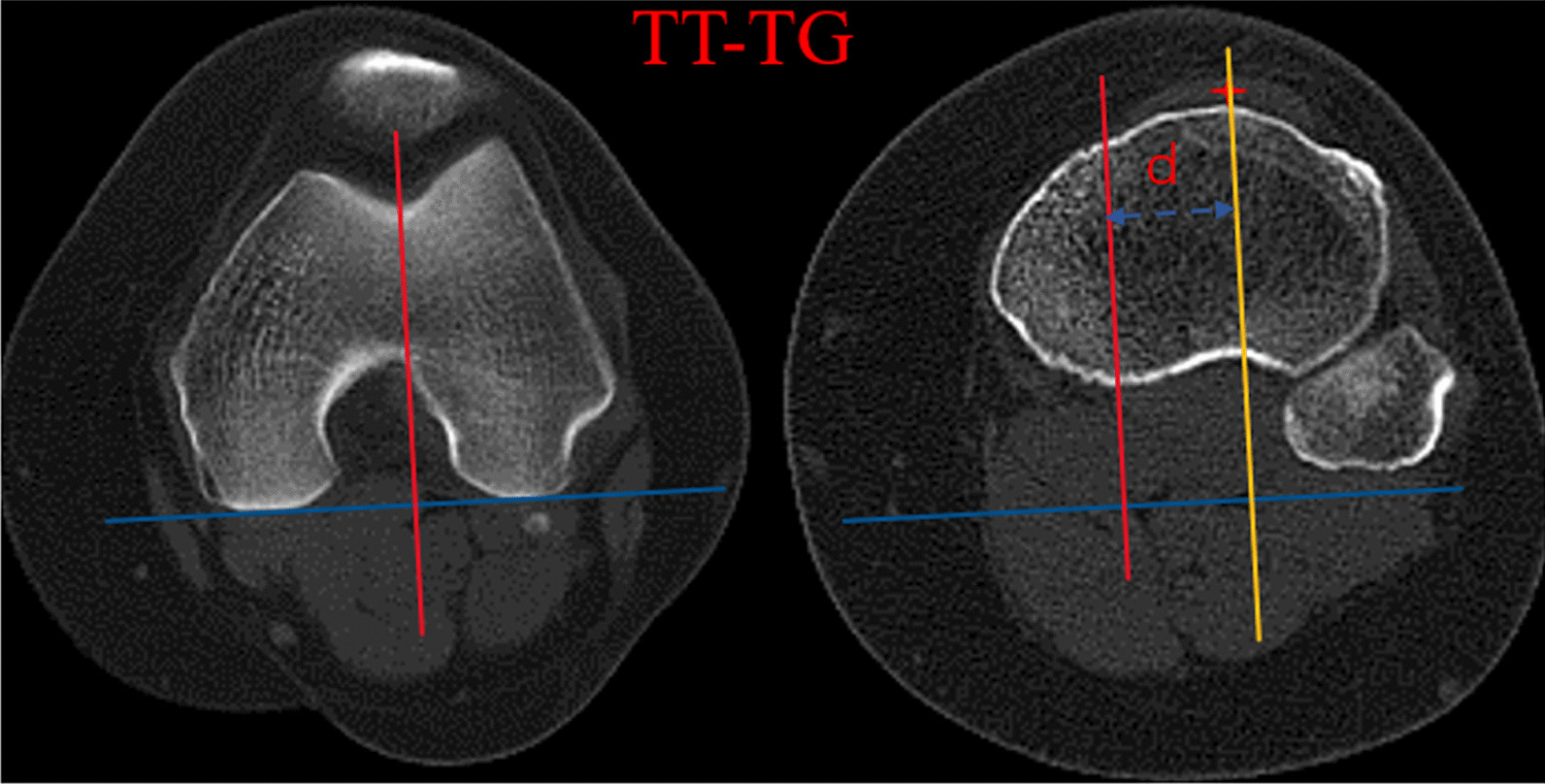
TT-TG, the posterior condylar line (blue line), was drawn tangential to the posterior condylar cortices at the level on which the posterior cortices of the femoral condyles were well confirmed. The second line (red line) was profiled from the deepest point of the trochlear groove perpendicular to the posterior condylar line. The third line (yellow line), passing through the midpoint of the patellar tendon attached at the tibial tubercle, was delineated paralleled to the second line. The TT-TG distance was presents as the blue dotted line. TT-TG, tibial tubercle-to-trochlear groove distance

The TT-TG distance was measured according to the technique described by Schoettle et al. [41]. The posterior condylar line was first drawn tangential to the posterior condylar cortices at the level on which the posterior cortices of the femoral condyles were well confirmed. A second line was profiled from the deepest point of the trochlear groove perpendicular to the posterior condylar line. The aforementioned two lines were maintained while scrolling inferiorly to the level of tibial tubercle. A third line, passing through the midpoint of the patellar tendon attached at the tibial tubercle, was delineated paralleled to the second line. The TT-TG distance was calculated as the linear distance between two paralleled lines (Fig. 2).

**Fig. 3:**
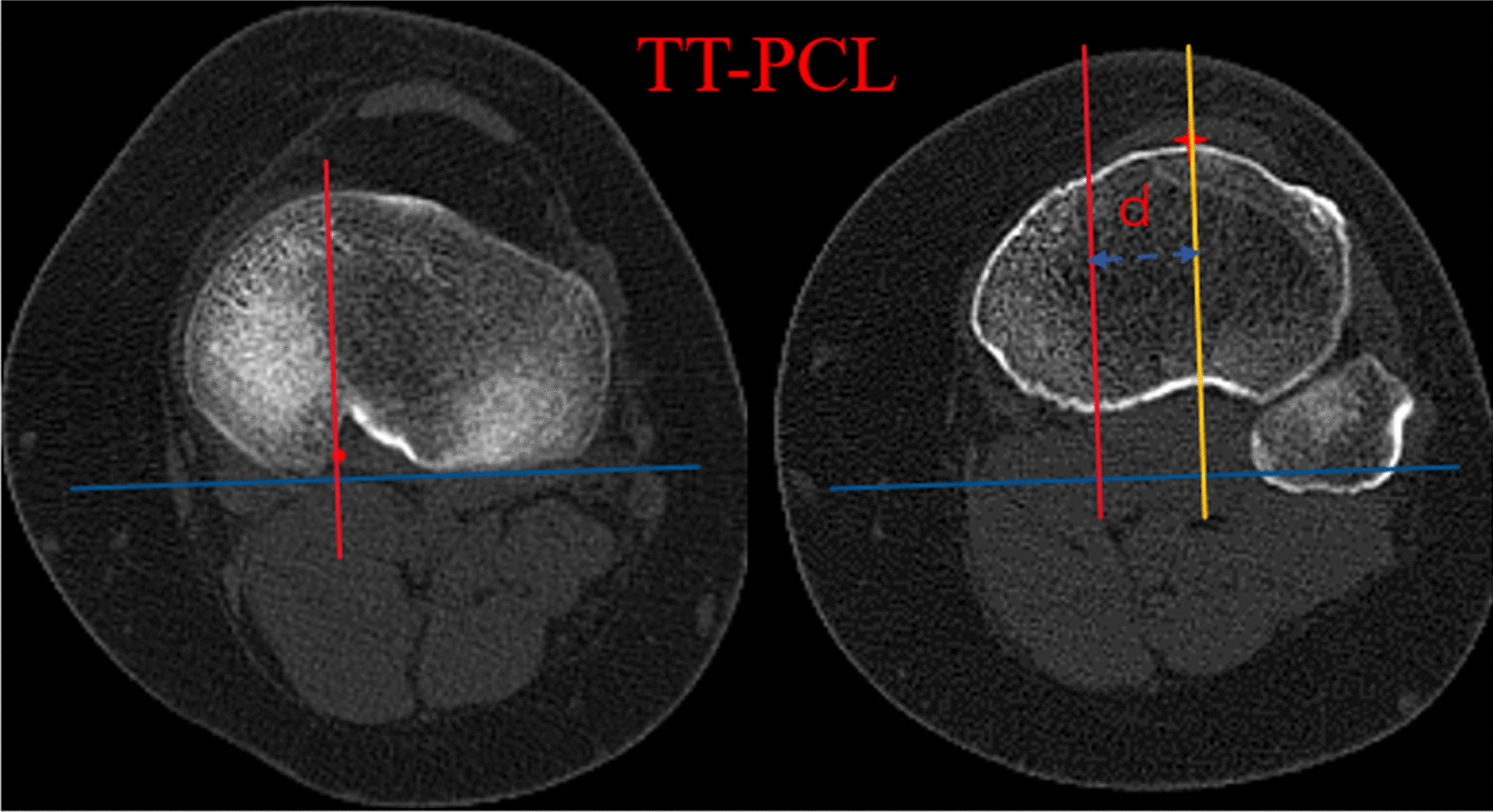
TT-PCL, the TT-PCL distance was determined as the mediolateral distance (blue dotted line) between one line (yellow line), passing the midpoint of the patellar tendon attached at the tibial tubercle (red asterisk) and perpendicular to the tibial posterior condylar line (blue line), and the other line (red line), passing the medial border of the PCL (red dot) and perpendicular to the tibial posterior condylar line (blue line). TT-PCL, tibial tubercle to posterior cruciate ligament; PCL, posterior cruciate ligament

Two aforementioned levels, containing the tibial tubercle and the PCL, were selected to calculate the TT-PCL distance. The TT-PCL distance was determined as the mediolateral distance between one line, passing the midpoint of the patellar tendon attached at the tibial tubercle and perpendicular to the tibial posterior condylar line, and the other line, passing the medial border of the PCL and perpendicular to the tibial posterior condylar line [42] (Fig. 3).

**Fig. 4:**
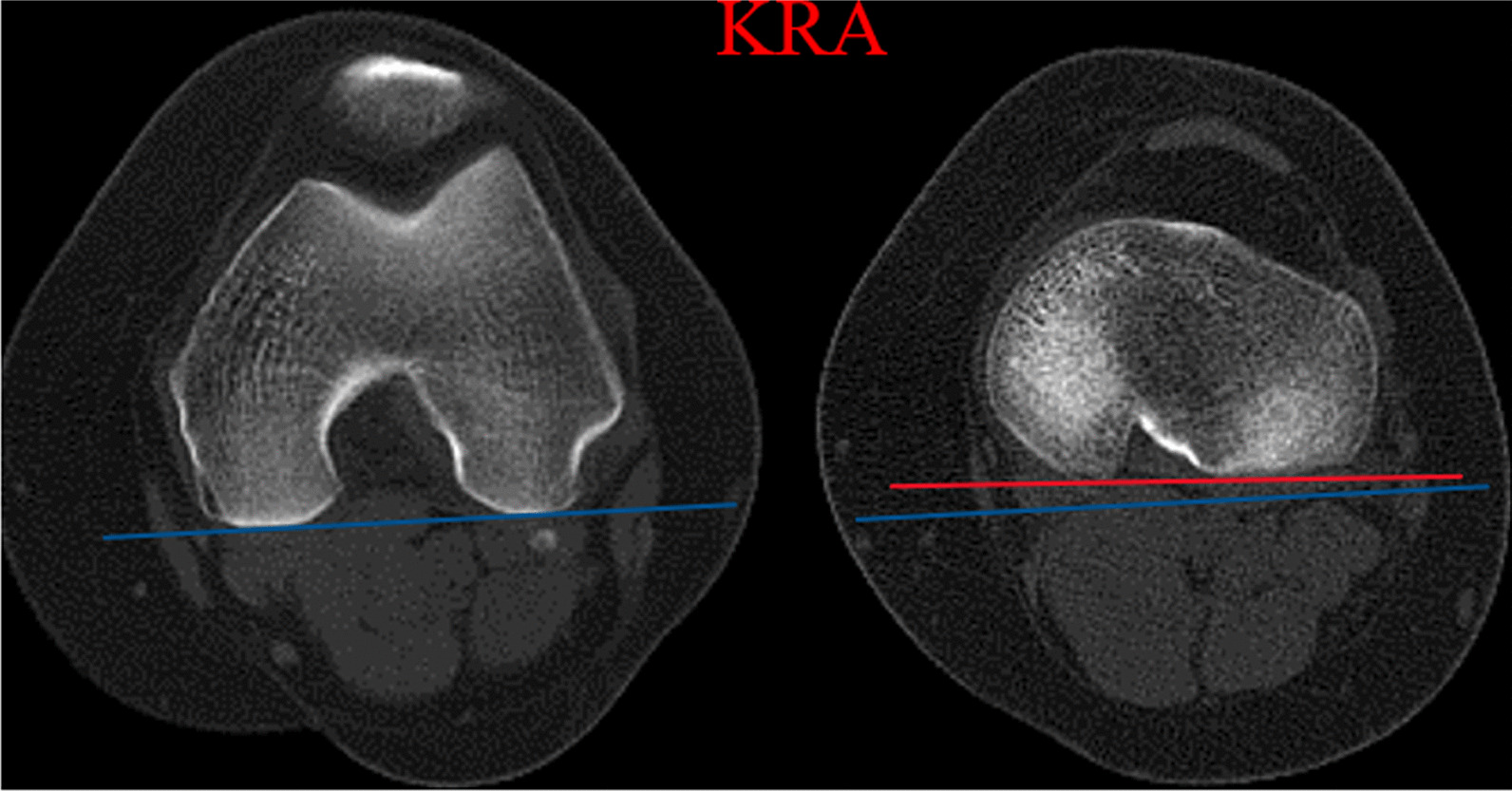
KRA, defined as the angle between the femoral posterior condylar line (blue line) and the tibial posterior condylar line (red line). KRA, knee rotation angle

The KRA represented relative rotation of the femur and tibia in relation to each other and was defined as the angle between the posterior condylar line of the femur and a tibial posterior condylar line (Fig. 4).

**Fig. 5:**
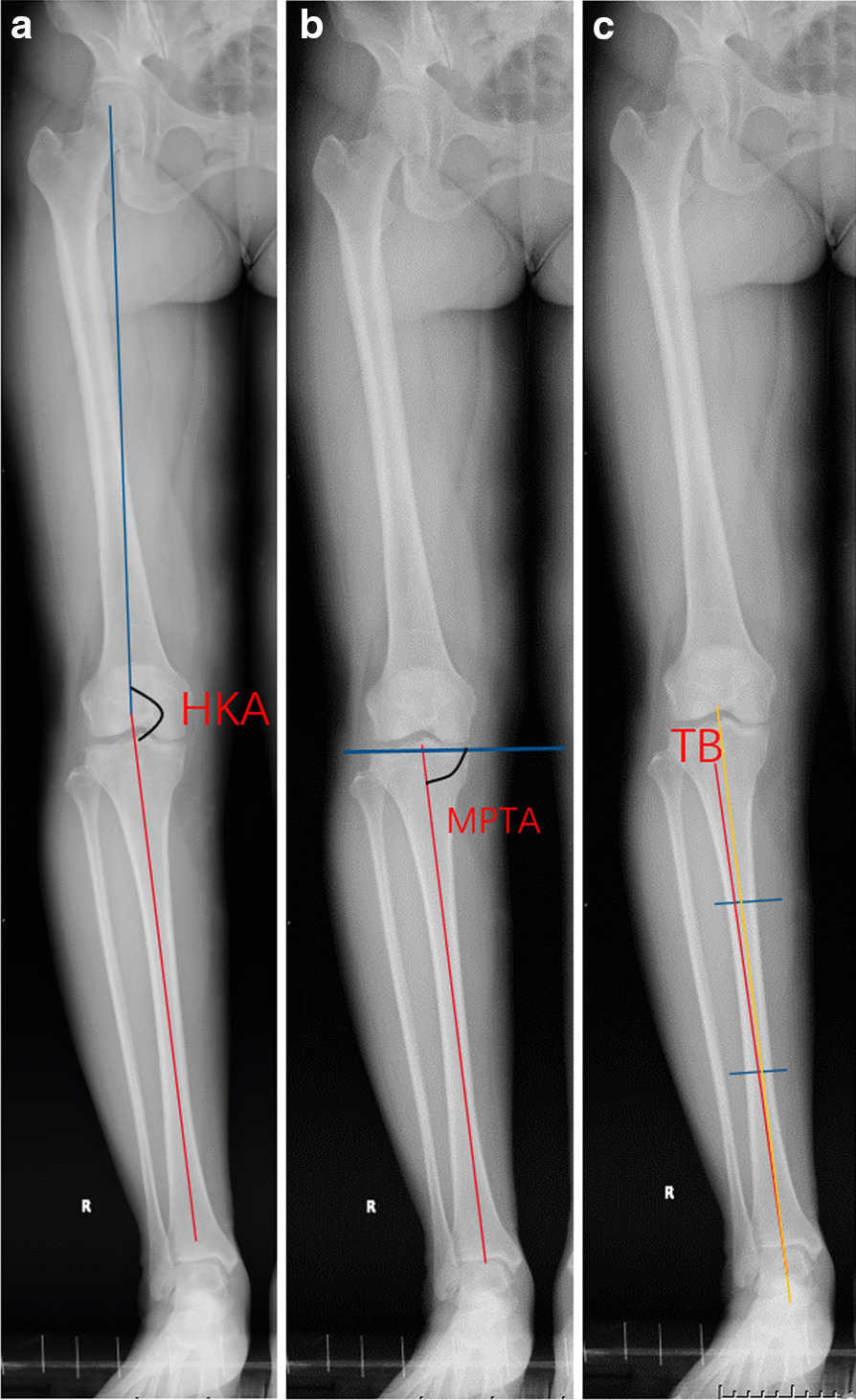
**a** HKA, formed by the mechanical axis of the femur (connecting the center of the femoral head and the center of the knee, blue line) and the mechanical axis of the tibia (connecting the center of the ankle and the center of the knee, red line). HKA, hip–knee–ankle angle. **b** MPTA, formed by the tibial mechanical axis (connecting the center of the ankle and the center of the knee, red line) and the line tangential to the proximal tibial articular surface (blue line). MPTA, medial proximal tibial angle. **c** TB, determined as the angle between the proximal third (yellow line) and distal third (blue line) mid-shaft axis. TB, tibial bowing

The HKA was formed by the mechanical axis of the femur (connecting the center of the femoral head and the center of the knee) and the mechanical axis of the tibia (connecting the center of the ankle and the center of the knee). A positive value indicates a genu varum (Fig. 5a). The MPTA was defined as the medial angle between the tibial mechanical axis and the line tangential to the proximal tibial articular surface (Fig. 5b). The TB was determined as the angle between the proximal third and distal third mid-shaft axis (Fig. 5c).

### Statistical considerations

Descriptive analyses were performed using frequencies with percentages for discrete or dichotomous variables as well as means with standard deviations (SDs) for continuous variables. The Kolmogorov–Smirnov normality test was performed to test whether our data fit a Gaussian distribution and all data passed the test. Pearson’s product moment correlation coefficients were initially calculated to assess the relationship between the discrepancies of the Akagi line and predictors, including sex, BMI, affected sides, age at surgery, and the defined imaging parameters. Subsequently, variables with a P value below 0.2 or a correlation coefficient of ≥ 0.25 were entered into a stepwise multivariable linear regression analysis. Model fit of the final selected model was assessed via normal quantile–quantile plots of the standardized residuals. The intra-observer and interobserver reliability were determined by calculating the interclass correlation coefficient (ICC), and an agreement of > 0.75 was considered excellent. All analyses were conducted using the IBM SPSS Statistics version 22.0 software package (IBM Inc., Chicago, IL, USA). All statistical tests were two-tailed, and a *p* value < 0.05 denoted a statistically significant difference. To detect an expected Pearson correlation magnitude of 0.25 (*α* = 0.05, *β* = 0.80), a sample size of 112 subjects was necessary.

## Results

Ultimately, baseline demographics and defined measurements were available for 140 patients (Table 1). No gender differences were found with respect to the angle *α* or angle *β*. All defined measurements were showed in Table 2 with excellent intra- and interobserver reliability.

**Table1:** Basic demographics

No	Sex (M)	Side (R)	Age	BMI
140	68	75	67 ± 6	23.89 ± 2.58

**Table 2:** Results of measurements

	Angle *α*	Angel *β*	TT-PCL	TT-TG	MPTA	HKA	TB	KRA
Mean (SD)	− 1.03 ± 4.25	7.93 ± 5.36	16.06 ± 6.24	9.31 ± 4.21	84.4 ± 3.1	5.61 ± 4.95	2.58 ± 1.46	4.60 ± 2.30
95%CI	− 1.74, − 0.32	7.03,8.22	15.01,17.09	8.60,10.01	83.89,84.92	4.78,6.43	2.36,2.83	4.22,4.98
Range	− 11.7, 8.7	− 6.9, 21.4	3, 35.7	1.3, 21.7	76.7, 92.6	− 8.4, 14.9	0.1, 6.4	0.1, 12
ICC (intra-, inter-)	0.91,0.89	0.87,0.84	0.93,0.90	0.85,0.79	0.90,0.91	0.97.0.95	0.89,0.86	0.95,0.97

**Fig. 6:**
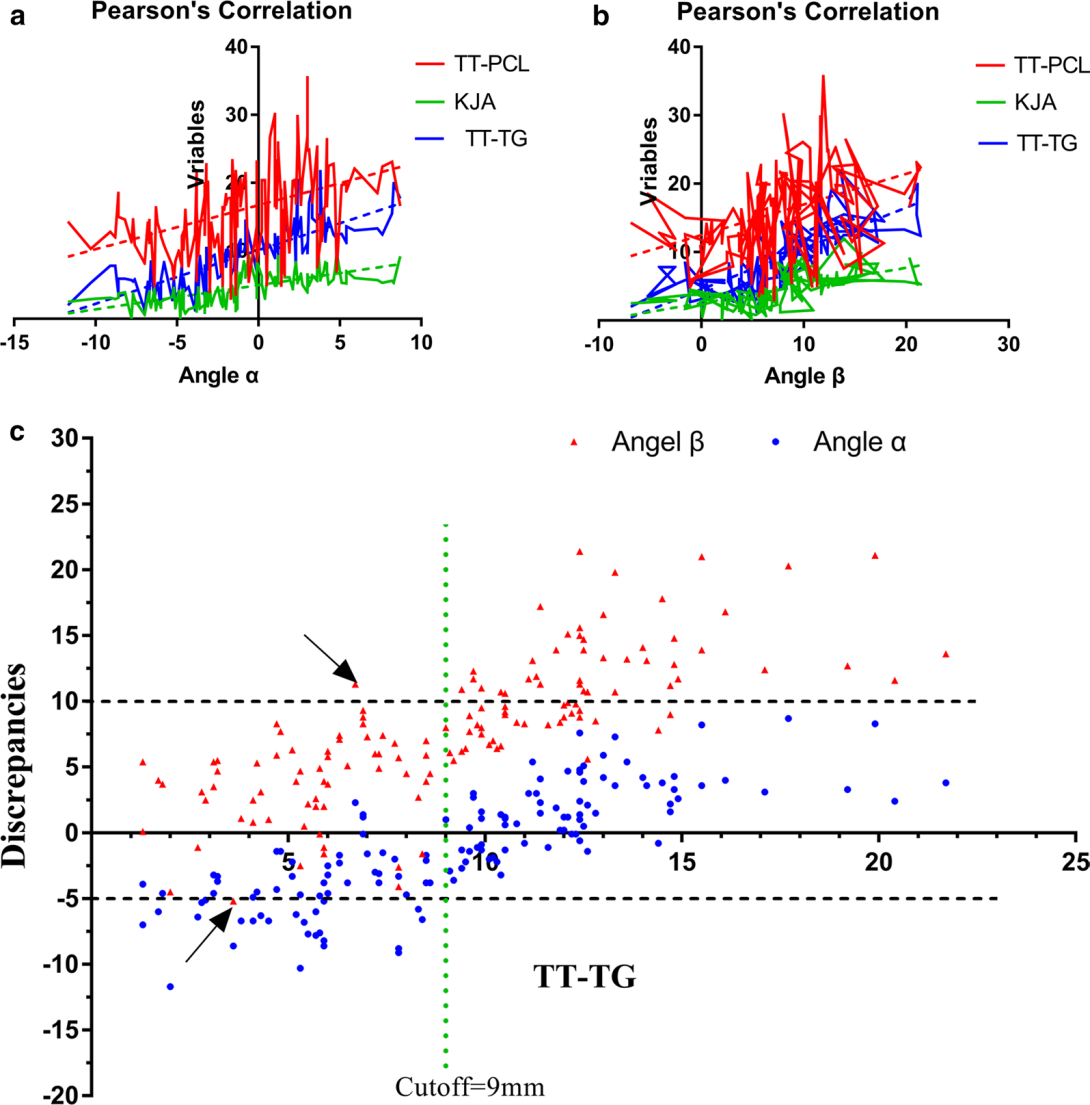
**a** Outcomes of Pearson’s correlation between angle *α* and variables. **b** Outcomes of Pearson’s correlation between angle *β* and variables. **c** Distribution of the angle *α* and the angle *β* based on the values of TT-TG. TT-TG, tibial tubercle-to-trochlear groove distance

The Akagi line rotated slightly internally in regard to the tibial AP axis (angle *α* = − 1.03 ± 4.25). Three variables, including TT-TG, TT-PCL, and KRA, tended to be positively correlated with the angle *α* (Table 3, Fig. 6a) and then were entered into a multivariable linear regression model. This model was significant (*F* = 114.1, *P* < 0.01), without multicollinearity (VIF < 10, Tolerance > 1). The regression coefficient were -10.83 of the constant, 0.54 for the TT-TG, 0.13 for the TT-PCL, and 0.57 for the KRA, and the *R*^2^ values and adjusted *R*^2^ values were 0.716 and 0.709, respectively. For the prediction of the angle *α*, a formula could be expressed as: Angle *α* (degrees) = − 10.83 + 0.54 × TT-TG + 0.13 × TT-PCL + 0.57 × KRA (Table 4).

**Table 3:** Results of Pearson’s correlation

	TT-PCL	TT-TG	KRA	MPTA	HKA	TB
Angle *α*	*p* < 0.01 *r* = 0.441	*p* < 0.01 *r* = 0.797	*p* < 0.01 *r* = 0.659	*P* = 0.46 *r* = 0.063	*P* = 0.79 *r* = 0.023	*P* = 0.42 *r* = − 0.107
Angel *β*	*p* < 0.01 *r* = 0.384	*p* < 0.01 *r* = 0.761	*p* < 0.01 *r* = 0.601	*P* = 0.84 *r* = 0.017	*P* = 0.87 *r* = 0.014	*P* = 0.67 *r* = − 0.036

**Table 4:** Results of multivariable linear regression

	Angle *α*		Angle *β*	
	*B*	*P*	*B*	*P*
Constant	− 10.83	0.000	− 3.22	0.001
TT-TG	0.54	0.000	0.72	0.000
TT-PCL	0.13	0.000	0.12	0.017
KRA	0.57	0.000	0.56	0.000
	*R*^2^ = 0.716, Adjusted *R*^2^ = 0.709		*R*^2^ = 0.626, Adjusted *R*^2^ = 0.618	

The Insall line rotated externally in regard to the tibial AP axis with a degree of 7.93 ± 5.36. The same three variables tended to be positively correlated with the angle *β* (Table 3, Fig. 6b) and then were entered into a multivariable linear regression model. This model was significant (*F* = 75.8, *P* < 0.01), without multicollinearity (VIF < 10, Tolerance > 1). The regression coefficient were − 3.22 of the constant, 0.72 for the TT-TG, 0.12 for the TT-PCL, and 0.56 for the KRA, and the *R*^2^ values and adjusted *R*^2^ values were 0.626 and 0.618, respectively. For the prediction of the angle *β*, a formula could be expressed as: Angle *β* (degrees) = − 3.22 + 0.72 × TT-TG + 0.12 × TT-PCL + 0.56 × KRA (Table 4).

According to the results of Pearson correlation, the TT-TG was strongly relative to the angle *α* and the angle *β*. Based on an assumed TT-TG cutoff of 9 mm and an acceptable rotational discrepancies (− 5 to 10), there were no outliers to use the Akagi line when the TT-TG > 9 mm, and were two outliers to use the Insall line when the TT-TG < 9 mm (Fig. 6c).

## Discussion

The present study demonstrated several main interesting findings: (1) The tibial tubercle, referring the Akagi line and Insall line, could be a reliable anatomic landmark for aligning the tibial component rotation with promising accuracy and excellent reproducibility. (2) Independent variables, including TT-TG, TT-PCL, and KRA, were found to be risk contributors for the angle *α* and the angle *β*. Two formulae were established via multivariable linear regression models. (3) It is advisable to determine the tibial component rotation according to the combination of the Akagi line and Insall line with a cutoff of TT-TG = 9 mm (Akagi line when TT-TG ≥ 9 mm, Insall line when TT-TG < 9 mm).

The axial rotation alignment, namely the reciprocal internal–external rotation alignment between the femoral and tibial components on their respective bony articular surface, deserves as a crucial requirement for successful TKA. High rates of discrepancies have been reported to be associated with the postoperative rotational alignment of tibial baseplate [24, 32]. Rotational mismatch, particularly excessive internal rotation, of tibial tray is suggested to be a risk factor of suboptimal outcomes following TKA. Panni et al. reviewed five studies and concluded that excessive internal rotation, commonly surpassing 10°, of the tibial tray could cause postoperative pain and compromise functional outcomes after TKA, while external rotation was not found to be related to the inferior results [37]. In this study, we established a relatively conservative acceptable rotation range from internal 5° to external 10°. Dalury et al. showed that internal or external tibial component rotation was implicated in impingement on the polyethylene, which could decrease range of motion and accelerate wear of ultra-high molecular weight polyethylene [13]. The mechanism underlying these detrimental consequences may be explained by the increased biomechanical forces induced by anteroposterior translation [45]. Unlike the femur, where have been verified that aligning the component parallel to the TEA could obtain optimum patellofemoral tracking and could minimize femorotibial wear motion and instability [9, 12, 15, 31], aligning the tibial tray rotation during TKA still remains a challenge. Numerous studies have proposed various anatomic landmarks of projected axes for guiding the tibial tray rotation, including the tibial tubercle, the mid-sulcus of the tibial spine, the posterior tibial condylar line, the tibial transcondylar line, the axis of the second metatarsus bone, and the transmalleolar ankle axis. However, there are no consensus on these defined references of which is most reliable to determine the rotational direction of the tibial component. The axis of the second metatarsus bone and the transmalleolar ankle axis would be susceptible to deformities caused by arthritis or trauma and positioning of ankle and foot. In addition, excessive external malrotation with substantial individual variations of the axis of the second metatarsus bone and the transmalleolar ankle axis have been reported with respect to the tibial AP axis [3, 22]. Concerning the mid-sulcus of the tibial spine, the posterior tibial condylar line, the tibial transcondylar line, osteophyte formation, and bone loss of the tibial articular surface often make it difficult to correctly identify these anatomic landmarks in an operating field. The tibial tubercle, commonly in terms of the Akagi line and Insall line, has been extensively investigated and widely accepted as a useful reference for tibial component rotation [38, 39].

According to the results, the Akagi line is approximately parallel to the tibial AP axis with slight internal rotation. The distributions of the angle *α* values indicate that using the Akagi line as rotational reference makes all cases within the defined safe zone when TT-TG distance ≥ 9 mm. On the other hand, using the Insall line as rotational reference makes 97% (75 in 77) cases within the defined safe zone when TT-TG distance < 9 mm. The effect, reported by Howell et al. [19], of the location of the tibial tubercle on the rotational alignment of the tibial component when using the Akagi line and Insall line partly matched the present study. The Akagi line, primarily depicted by Akagi et al. in 2004, was considered to be perpendicular to the projected axis of the TEA and was reconfirmed with an angle of 0.2° ± 2.8° in relation to the tibial AP axis [3, 4]. Noteworthily, the two studies were both conducted in healthy population, and then, similar results were found in OA patients by Kim et al. [23] and Kim et al. [22] who also reported that TB had no influence on the results. The same goes for our study that coronal alignment of lower extremity does not impact the angle *α* and angle *β*. There were else literature demonstrating that the Akagi line rotated mildly externally relative to the tibial AP axis [1, 27]. The Insall line, taking the medial third of the tibial tubercle as anterior landmark, has been widely used in clinical practice, although it has been proved to be in external rotation with regard to the tibial AP axis [4, 23, 27]. A slight external of tibial tray is usually accepted or even preferred by surgeons, considering that it benefits patellofemoral tracking [50]. Lützner et al. recommend to align rotational orientation of tibial tray using the medial third rather than the medial border of the tibial tubercle [26]. In contrast to our results that no correlations exist between the mechanical alignment of lower extremity and the two defined angles, several studies detected a significant difference between varus and vulgus knee.

Recently, several novel references have been put forward and been suggested to be used to determine the rotation alignment of the tibial component [7, 23]. The curve-on-curve technique, using the anterior tibial curved cortex as the rotational reference, was reported to be more reliable than the tibial tubercle for the rotational alignment of the tibial component [7, 23]. Baldini et al. suggested an ATR (after tibial resection) line passing through the lateral edge of PCL at its tibial attachment after resection and the most prominent point of the tibial tubercle, and reported that the line was located between the Akagi line and Insall line [7]. Kawahara et al. advised to take medial sixth of the tibial tubercle as the anterior anatomic landmark [21]. Yet, these references have not been widely used or accepted possibly due to low practicality or vulnerability to shape variability and asymmetrical morphology.

To avoid depending on anatomic landmarks, the ROM (range of motion) technique, which allowed the tibial baseplate to float into the orientation with respect to the femoral component while the knee was placed through a full arc of motion, was preferred [13, 16, 17]. What calls for special attention is that the technique is experience-required and tends to rotate the tibial baseplate externally [5, 11, 20]. Computer-assisted TKA or patient-specific instrumentation may improve the positioning accuracy of the tibial tray, but then, they are more expensive than conventional instruments, and are present with a learning curve [8, 11]. When performing a TKA, surgeons may be faced with a dilemma: how to compromise between bone coverage and rotation alignment of tibial tray. Martin et al. found that maxing tibial coverage, in particular with symmetrical designs, was prone to cause internal malrotation of tibial component. There are several limitations to the current study. Firstly, only Chinese population were recruited in our investigation, therefore, there might be anatomic differences from the Caucasian population [18, 46]. Notably, so far, most similar researches were established in Asian region. There is a requirement of more information and studies focusing on other race. Secondly, the applicability of this study may be restricted in the condition that the center of the PCL is difficult to clearly identify when choosing a posterior-stable prosthesis. Thirdly, although we tried to keep all subjects in the same fixed position, the inevitable internal or external rotation of lower extremity and slight knee flexion could have a detrimental effect on the reciprocal position between the femur and tibial.

## Data Availability

The data used for analysis in our study are available from the corresponding author on reasonable request.

## Abbreviations

AP: Anteroposterior
TT-TG: Tibial tubercle-to-trochlear groove distance
TT-PCL: Tibial tubercle to posterior cruciate ligament
KRA: Knee rotation angle
HKA: Hip–knee–ankle angle
MPTA: Medial proximal tibial angle
TB: Tibial bowing
TKA: Total knee arthroplasty
TEA: Trans-epicondylar axis
PCL: Posterior cruciate ligament
SDs: Standard deviations
ICC: Interclass correlation coefficient
